# In silico analysis as a strategy to identify candidate epitopes with human IgG reactivity to study *Porphyromonas gingivalis* virulence factors

**DOI:** 10.1186/s13568-019-0757-x

**Published:** 2019-03-11

**Authors:** Ellen Karla Nobre dos Santos-Lima, Kizzes Araújo Paiva Andrade Cardoso, Patrícia Mares de Miranda, Ana Carla Montino Pimentel, Paulo Cirino de Carvalho-Filho, Yuri Andrade de Oliveira, Lília Ferreira de Moura-Costa, Teresa Olczak, Isaac Suzart Gomes-Filho, Roberto José Meyer, Márcia Tosta Xavier, Soraya Castro Trindade

**Affiliations:** 1 Postgraduate Program in Immunology, Federal University of Bahia, Salvador, Bahia Brazil; 2 Biotechnology Postgraduate Program, Federal University of Bahia, Salvador, Bahia Brazil; 3 Bahian School of Medicine and Public Health, Salvador, Bahia Brazil; 4 Dentistry Course, Feira de Santana State University, Feira de Santana, Bahia Brazil; 5 Department of Biointeraction, Federal University of Bahia, Salvador, Bahia Brazil; 6 Faculty of Biotechnology, University of Wrocław, Wrocław, Lower Silesia Poland; 7 Department of Health, Feira de Santana State University, Avenida Transnordestina s/n, Novo Horizonte, Feira de Santana, Bahia CEP 44036-900 Brazil

**Keywords:** Immune response, Immunoinformatics, Lys-gingipain, Neuraminidase, Periodontitis, Sialidase

## Abstract

*Porphyromonas gingivalis* (Pg) is one of the main pathogens in chronic periodontitis (CP). Studies on the immunogenicity of its virulence factors may contribute to understanding the host response to infection. The present study aimed to use in silico analysis as a tool to identify epitopes from Lys-gingipain (Kgp) and neuraminidase virulence factors of the Pg ATCC 33277 strain. Protein sequences were obtained from the NCBI Protein Database and they were scanned for amino acid patterns indicative of MHC II binding using the MHC-II Binding Predictions tool from the Immune Epitope Database (IEDB). Peptides from different regions of the proteins were chemically synthesized and tested by the indirect ELISA method to verify IgG immunoreactivity in serum of subjects with CP and without periodontitis (WP). T cell epitope prediction resulted in 16 peptide sequences from Kgp and 18 peptide sequences from neuraminidase. All tested Kgp peptides exhibited IgG immunoreactivity whereas tested neuraminidase peptides presented low IgG immunoreactivity. Thus, the IgG reactivity to Kgp protein could be reaffirmed and the low IgG reactivity to Pg neuraminidase could be suggested. The novel peptide epitopes from Pg were useful to evaluate its immunoreactivity based on the IgG-mediated host response. In silico analysis was useful for preselecting epitopes for immune response studies in CP.

**Electronic supplementary material:**

The online version of this article (10.1186/s13568-019-0757-x) contains supplementary material, which is available to authorized users.

## Introduction

Chronic periodontitis is a multifactorial and polymicrobial disease, which may negatively influence systemic diseases (Hajishengallis 2015). Its pathogenesis is related to host immune inflammatory factors and to a synergistic and dysbiotic oral microbiome (Hajishengallis et al. 2012; Hajishengallis 2014; Hajishengallis and Lamont 2014). In light of the diversity of the human oral microbiome (Proctor et al. 2018), immunoinformatics brings tools that provide faster analysis of virulence factors, considering the polymicrobial character of chronic periodontitis, and contribute to understanding the interaction between the oral microbiome and the host.

*Porphyromonas gingivalis*, a keystone pathogen related to periodontal dysbiosis (Hajishengallis 2014), produces various virulence factors that can act as immunogenic molecules. The host response to infection may be better understood by progressively investigating the immunogenicity of* P. gingivalis* virulence factors (Trindade et al. 2008, 2012a, b, 2013; Olczak et al. 2010; Bengtsson et al. 2015; Carvalho-Filho et al. 2016; Tada et al. 2016; Dashper et al. 2017).

Gingipains (cysteine proteases) are the main proteases of *P. gingivalis* (Guo et al. 2010), one of their main functions being heme acquisition (Smalley and Olczak 2017). These proteases are also immunogenic proteins, contributing to the pathogen’s ability to induce chronic periodontitis, since they can elicit a humoral immune response in humans, inducing higher serum levels of specific IgG in individuals with chronic periodontitis (O’Brien-Simpson et al. 2000; Inagaki et al. 2003; Nguyen et al. 2004).

Lys-gingipain (Kgp) is considered a major virulence factor of *P. gingivalis* (de Diego et al. 2014) and is also involved in the bacteria-host interaction through the production of cytokines, such as IL-17A and INF-γ (Bittner-Eddy et al. 2013). Kgp (1723 aa/187 kDa) is cleaved into four chains: the catalytic subunit and three adhesion domains (UniProt B2RLK2).

Although not as well studied as Kgp, neuraminidase (sialidase) of *P. gingivalis* is secreted by the microorganism for neuraminic acid (sialic acid) acquisition from sialoglycoconjugates of the host. Sialic acids are incorporated into the microorganism structures, thus mimicking the host cell and confounding the immune response, mainly under stressful microenvironmental conditions (Li et al. 2012; Xu et al. 2017). Sialidase deficiency in *P. gingivalis* increases sensitivity to hydrogen peroxide, decreases resistance to the action of complement and reduces virulence after subcutaneous injection in mice, likely by influencing capsule biosynthesis (Moncla et al. 1990; Aruni et al. 2011; Li et al. 2012; Xu et al. 2017).

Moreover, the sialidase activity may be involved in the production, maturation and secretion of gingipains and other virulence factors of *P. gingivalis*, probably due to their sialylation (glycosylation) (Aruni et al. 2011; Xu et al. 2017). Importantly, inhibitors of these two virulence factors studied herein could be promising for use in the treatment of chronic periodontitis and associated systemic diseases (Cueno et al. 2014; Olsen and Potempa 2014; Inaba et al. 2016; Xu et al. 2017).

The present study aimed to use in silico analysis as a strategy to identify potential immunogenic peptides from those *P. gingivalis* relevant proteins. Selected epitopes were evaluated concerning their immunoreactivity based on the IgG-mediated host response in order to contribute to immunogenicity studies of this keystone pathogen.

## Materials and methods

### Selection of subjects for immunoreactivity test of peptides

For sample size calculation, considering the human IgG levels against *P. gingivalis* extract (Trindade et al. 2008), the absorbance value of 133 was estimated as a relevant difference to be detected, with a standard deviation of 126. Therefore, 21 participants were estimated in each group, considering the significance level of 5%, the test power of 90%, and 10% increase to predict losses.

Participants were selected considering exclusion criteria: age less than 18 years, number of teeth less than 10, history of systemic diseases, current pregnancy, periodontal treatment performed up to 1 year before oral examination, current or former cigarette smoking habit, alcoholism, use of antibiotics and anti-inflammatories, respectively, at 6 and 2 months before the selection.

A structured questionnaire was applied to obtain information about individuals’ health conditions, and then periodontal clinical examination and collection of peripheral blood (5 mL) were performed. Chronic periodontitis was diagnosis followed the consensus of the American Academy of Periodontology (Armitage 1999; Lindhe et al. 1999; Caton et al. 2018) and subjects were classified according to the periodontal criteria proposed by Gomes-Filho et al. (2007). Participants were separated into two groups according to the periodontal diagnosis: a group with chronic periodontitis (CP) and a group without periodontitis (WP).

### Production of *P. gingivalis* extract

After anaerobic culture of bacteria, the immunogenic extract of *P. gingivalis* ATCC 33277 strain (NCBI Taxonomy ID: 431947) was produced according to the standardized protocol (Trindade et al. 2008). The total protein was measured, and the sonicated extract was stored at − 20 °C.

### Sera pools for immunoreactivity test of peptides

The indirect ELISA method was performed for IgG detection in each serum sample of selected individuals, and 5 µg/mL of *P. gingivalis* (Pg) extract was used as the antigen (Trindade et al. 2008). Two sera pools were obtained for use in the immunoreactivity test. The CP pool comprised serum samples from individuals with chronic periodontitis with the highest levels of anti-Pg IgG obtained among samples. The WP pool comprised serum samples from individuals without periodontitis with the lowest levels of anti-Pg IgG obtained among samples. 200 µL of each sample were pooled into CP and WP pools, homogenized and stored at − 20 °C.

### Peptide prediction

This in silico analysis assumed that CD4^+^ T cells mainly recognize short linear peptides from proteins produced by extracellular pathogens, such as *P. gingivalis*, presented by MHC class II molecules (Vyas et al. 2008; Rocha and Neefjes 2008). Protein sequences (YP_001929844/BAG34127.1) were obtained from the Protein Database of the National Center for Biotechnology Information (NCBI), USA, and they were scanned for amino acid patterns indicative of MHC II binding using the MHC II Binding Prediction tool (http://tools.immuneepitope.org/mhcii/) from the Immune Epitope Database and Analysis Resource (IEDB), which is a previously validated method. This tool employs different methods to predict MHC class II epitopes, including a consensus approach (default) used herein (Wang et al. 2008, 2010; Vita et al. 2010).

This tool requires specification of the HLA allele/haplotype to make binding predictions; therefore the analysis considered 09 HLA alleles (loci DQ and DR), which were observed in the previous study involving subjects with chronic periodontitis from Salvador, Bahia, and Brazil (Monteiro et al. 2017). The same HLA alleles were used to predict Kgp and neuraminidase immunogenic peptides (Additional file 1: Table S1).

### Post-prediction analysis

Epitope Cluster Analysis (http://tools.iedb.org/cluster/) (Kim et al. 2012) was performed to group peptide sequences into clusters. Peptide sequences were compared to those in the IEDB by searching for similarity, using the Basic Local Alignment Search Tool (BLAST) (Altschul et al. 1990). Finally, the peptide sequences obtained were compared to current data published by the NCBI Protein Database (YP_001929844/BAG34127.1) to identify protein regions.

### Peptide synthesis

Peptides from different regions of the proteins were chemically synthesized by *AminoTech Pesquisa e Desenvolvimento LTDA*, Diadema, SP, Brazil, using the Fmoc strategy. Purification (95%) was achieved by reverse-phase high-performance liquid chromatography (HPLC) and the peptides were characterized by mass spectrometry by AminoTech. Then, freeze-dried peptides were solubilized in 0.5 M carbonate-bicarbonate buffer (pH 9.6) and stored at − 20 °C.

### IgG immunoreactivity test of peptides

Synthetic peptides were tested by the indirect ELISA method (Trindade et al. 2008) to verify the levels of IgG in CP and WP sera pools. 10 µg/mL of each peptide was used as an antigen and 5 µg/mL of *P. gingivalis* extract was used as a positive control. A checkerboard ELISA was performed to obtain the appropriate conditions for the equivalence zone in the antigen–antibody reaction, analyzing the best concentrations of antigen, serum and conjugate. For each combination (antigen, serum and conjugate), the coefficients of O.D. (optical density) between the CP pool and the WP pool were determined for each analyzed peptide. The coefficient expresses the difference of the mean IgG levels between the CP and the WP sera pools.

### Submission to the Immune Epitope Database

The tested peptides were submitted to the Immune Epitope Database and Analysis Resource (IEDB) through the data submission tool (DST)/Wizard submission method and they can be accessed at submission ID 1000760 to Kgp peptides and at ID 1000766 to neuraminidase peptides.

### Analysis of population coverage

A coverage analysis of published peptides was performed using the Population Coverage tool from IEDB “Class II separate” (http://tools.iedb.org/population/) (Bui et al. 2006), which uses the HLA allele genotypic frequencies of the Allele Frequency Net Database. At present, according to the Population Coverage Tutorial, the Allele Frequency Database provides allele frequencies for 115 countries and 21 different ethnicities grouped into 16 different geographical areas (http://tools.iedb.org/population/help/#population_info. Accessed 15 August 2018).

### Peptide presentation by protein modeling

The published peptides were schematically presented in their protein region after protein structure prediction. I-TASSER on-line server (http://zhanglab.ccmb.med.umich.edu/I-TASSER/) (Roy et al. 2010) and PyMOL 1.7.4.4 Edu software were used for modeling analysis. The sequence of each Kgp protein region and neuraminidase was obtained from the NCBI Protein Database annotation (YP_001929844 and BAG34127.1, respectively).

### Statistical analysis

Descriptive analysis was performed for the characterization of the groups. To compare the groups, the Mann–Whitney U test was used, based on the distribution assessed by the Kolmogorov–Smirnov test. Data related to the dichotomous variable were tested with Fisher’s exact test. For all statistical procedures a significance level of 5% was applied (P ≤ 0.05).

For screening of peptides, a checkerboard evaluation was performed and the coefficients of absorbance between CP and WP pools were determined (difference of the O.D. value between the sera pools). The nature of the data does not allow statistical analysis between the coefficients.

## Results

### Selection of subjects for immunoreactivity test of peptides

Forty-one participants who attended the School of Dentistry of the Feira de Santana State University, Bahia, Brazil, were enrolled. They were clinically classified into the CP group (20 subjects) and the WP group (21 subjects) according to periodontal parameters. The groups were homogeneous with respect to age, the proportion between female and male subjects, and teeth number (Table 1).

**Table 1:** Characteristics of subjects without periodontitis and patients with chronic periodontitis (Brazil, 2018)

Subjects	WP group^a^ (n = 21)	CP group^a^ (n = 20)	P-value (≤ 0.05)
*Age in years*	34.5	33.0	0.89
Median (IQR)	(24.5–41.3)	(24.8–45.0)	
*Sex proportion*	18/3	14/6	0.28
(female/male)			
*Number of teeth*	25.0	26.0	0.39
Median (IQR)	(20.5–27.0)	(20.3–28.0)	
*%BOP*	11.0	44.6	0.00
Median (IQR)	(4.9–22.9)	(28.4–59.4)	
*%CAL ≥ 3* *mm*	1.2	26.8	0.00
Median (IQR)	(0.0–14.8)	(8.8–45.5)	
*%CAL ≥ 5* *mm*	0.0	11.5	0.00
Median (IQR)	(0.0–0.0)	(3.5–29.1)	
*%PD ≥ 4* *mm*	0.0	15.3	0.00
Median (IQR)	(0.0–2.7)	(8.3–27.3)	

Third molars were excluded

Mann–Whitney U test and Fisher’s exact test

CP: chronic periodontitis; WP: without periodontitis; BOP: bleeding on probing; CAL: clinical attachment level; PD: probing depth; IQR: interquartile range

^a^Classification according to Gomes-Filho et al. (2007)

### Sera pools for immunoreactivity test

Forty-one serum samples were tested (anti-Pg IgG): 20 samples of the CP group and 21 samples of the WP group. Six samples were selected and pooled into the CP pool (O.D.: 0.53–1.10) and five samples were selected and pooled into the WP pool (O.D.: 0.22–0.38).

### Peptide prediction and post-prediction analysis

T-cell epitope prediction resulted in 16 peptide sequences from Kgp and 18 peptide sequences from neuraminidase: two predicted epitopes for each tested HLA allele, which had presented a lower percentile rank and had been located in two different regions of the analyzed protein (Tables 2, 3).

**Table 2:** Putative epitopes of Kgp obtained from the peptide prediction analysis of the protein sequence (Brazil, 2018)

Protein	HLA allele/haplotype	Peptide	Peptide sequence (15-mer)	Start	End
Kgp (1723 aa)	DQA1*05:01/DQB1*03:01	kgp1	ESFGLGGIGVLTPDN	1028	1042
	DQA1*05:01/DQB1*03:01	kgp2	LSPLRASNVAISYSS	1467	1481
	DQA1*05:01/DQB1*02:01	kgp3	TDMFYIDLDEVEIKA	1137	1151
	DQA1*05:01/DQB1*02:01	kgp4	GTAAADFEVIFEETM	1533	1547
	DQA1*01:02/DQB1*06:02	kgp5	MLGTMRGVRIAALTI	160	174
	DQA1*01:02/DQB1*06:02	kgp6	NVAISYSSLLQGQEY	1474	1488
	DRB3*01:01	kgp9	SDLNYILLDDIQFTM	1317	1331
	DRB3*01:01	kgp10	VLGIMIDDVVITGEG	1579	1593
	DRB1*13:02	kgp11	ANDVRANEAKVVLAA	733	747
	DRB1*13:02	kgp12	NYDVVITRSNYLPVI	661	675
	DRB1*15:01	kgp13	NYDVVITRSNYLPVI	661	675
	DRB1*15:01	kgp14	MRKLLLLIAASLLGV	1	15
	DRB1*07:01	kgp15	YVAFRHFQSTDMFYI	1128	1142
	DRB1*07:01	kgp16	PATGPLFTGTASSNL	773	787
	DRB5*01:01	kgp17	RMLVVAGAKFKEALK	243	257
	DRB5*01:01	kgp18	VPFVYNAAAYARKGF	136	150
	DRB4*01:01	kgp19	SDLNYILLDDIQFTM	1317	1331
	DRB4*01:01	kgp20	NYLPVIKQIQAGEPS	670	684

Start and end correspond to the positions in the Kgp sequence. For Kgp 9/19 and Kgp 12/13, only one sequence was considered

**Table 3:** Putative epitopes of neuraminidase obtained from the peptide prediction analysis of the protein sequence (Brazil, 2018)

Protein	HLA allele/haplotype	Peptide	Peptide sequence (15-mer)	Start	End
Neuraminidase (526 aa)	DQA1*05:01/DQB1*03:01	N1	LMIFVGGVGLWQSTP	268	282
	DQA1*05:01/DQB1*03:01	N2	RTTALSADSVAGRCF	116	130
	DQA1*05:01/DQB1*02:01	N3	DGTIGYFVEEDDEIS	496	510
	DQA1*05:01/DQB1*02:01	N4	SLVFIRFVLDDLFDA	510	524
	DQA1*01:02/DQB1*06:02	N5	VVFCCLMAMMHLSGQ	17	31
	DQA1*01:02/DQB1*06:02	N6	LKTANGTLIAMADRR	199	213
	DRB3*01:01	N7	YSDMTLLADGTIGYF	488	502
	DRB3*01:01	N8	PDYKGRVSYDSFPIS	98	112
	DRB4*01:01	N9	ALYLLVSDSLAVRDL	83	97
	DRB4*01:01	N10	LMAMMHLSGQEVTMW	22	36
	DRB5*01:01	N11	KQIRIGFSLPKETEE	65	79
	DRB5*01:01	N12	KTANGTLIAMADRRK	200	214
	DRB1*13:02	N13	IPSILKTANGTLIAM	195	209
	DRB1*13:02	N14	VALVQTQAGKLLMIF	257	271
	DRB1*15:01	N15	QAGKLLMIFVGGVGL	263	277
	DRB1*15:01	N16	PASRRLYREYEALFV	170	184
	DRB1*07:01	N17	FGDVALVQTQAGKLL	254	268
	DRB1*07:01	N18	YRIPSILKTANGTLI	193	207

Start and end correspond to the positions in the protein sequence

There was no sequence cluster formation (identity threshold 90%) for 16 peptide sequences from Kgp and for 18 peptide sequences from neuraminidase, which indicates that all sequences obtained are different from each other.

There was also no exact similarity (exact matches—100%) when comparing peptide sequences with peptides published in IEDB, which indicated that those sequences had not been published yet by another research group.

The peptide sequences were compared to current data published by the NCBI Protein Database (YP_001929844/BAG34127.1) to identify protein regions (Additional file 1: Tables S2, S3).

In addition, Kgp14 was not synthesized due to its position in the protein (1–15), which in principle should no longer be present in the mature protein.

### IgG immunoreactivity of peptides

Nine predicted peptides from Kgp (Kgp1, 6, 11, 12, 15, 16, 17, 18 and 20) and eight peptides from neuraminidase (N5, 6, 10, 11, 15, 16, 17 and 18) were selected for synthesis and immunoreactivity analysis.

**Fig. 1:**
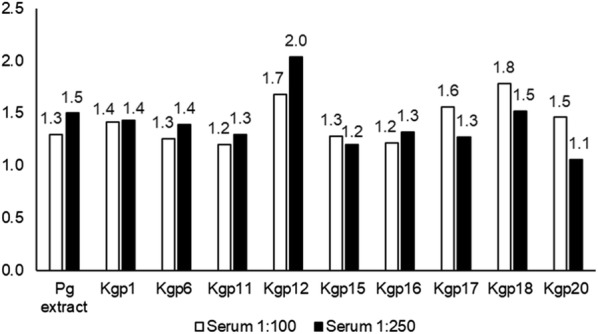
Coefficients of absorbance between CP sera pool and WP sera pool after checkerboard ELISA analysis of Kgp synthetic peptides. Brazil, 2018. CP: chronic periodontitis; WP: without periodontitis. The coefficient is the difference of the O.D. value between the sera pools, and it expresses the difference of the mean IgG levels between the CP and the WP sera pools. *P. gingivalis* (Pg) extract and peptides were used as the antigen in the indirect ELISA test. 1:100 and 1:250 serum concentrations are represented. Kgp12 presented a higher coefficient between CP and WP sera pools in 1:250 serum concentration. Checkerboard ELISA best acquired condition: 5 µg/mL Pg extract concentration, 10 µg/mL peptide concentration, 1:250 sera pool concentration and 1:25,000 anti-human IgG peroxidase conjugate concentration. The nature of the data does not allow statistical analysis between the coefficients

Kgp synthetic peptides presented immunoreactivity to IgG. Kgp12, Kgp18 and Kgp17, respectively, represented a higher coefficient between CP and WP sera pools, so they were selected for subsequent assays. A greater difference between the absorbance values of the CP and WP sera pools was observed when the Kgp12 was tested in the following conditions: 10 µg/mL peptide concentration, 1:250 sera pool concentration and 1:25,000 anti-human IgG peroxidase conjugate concentration (Checkerboard ELISA best acquired condition) (Fig. 1).

Low levels of IgG were observed for each neuraminidase synthetic peptides tested. The IgG reactivity of the CP sera pool was similar to the WP sera pool for all of the peptides and it could not be compared to the immunoreactivity observed when the *P. gingivalis* extract was used as an antigen (Additional file 1: Figure S1).

In this immunoreactivity analysis, all HLA alleles tested with neuraminidase peptides were also tested with Kgp peptides. DQB1*03:01 and DRB1*13:02 were tested only with Kgp peptides (Additional file 1: Table S4).

### Submission to the Immune Epitope Database

Tested peptides were deposited in the IEDB and they are available for public visualization: Kgp reference ID 1032999 and neuraminidase reference ID 1033135 (Table 4).

**Table 4:** Immune Epitope Database (IEDB) identifiers of the tested peptides (Brazil, 2018)

Peptide	IEDB Epitope ID	IEDB Assay ID
Kgp1	759539	3817487
Kgp6	763531	3817488
Kgp11	758136	3817486
Kgp12	763561	3817485
Kgp15	766856	3817489
Kgp16	763583	3817492
Kgp17	764357	3817490
Kgp18	766224	3817493
Kgp20	763564	3817491
N5	767013	3838946
N6	767000	3838952
N10	767001	3838947
N11	766996	3838948
N15	767006	3838953
N16	767004	3838949
N17	766988	3838951
N18	767015	3838950

### Population coverage

The result of the population coverage analysis is shown in Additional file 1: Tables S5, S6.

### Peptide presentation by protein modeling

**Fig. 2:**
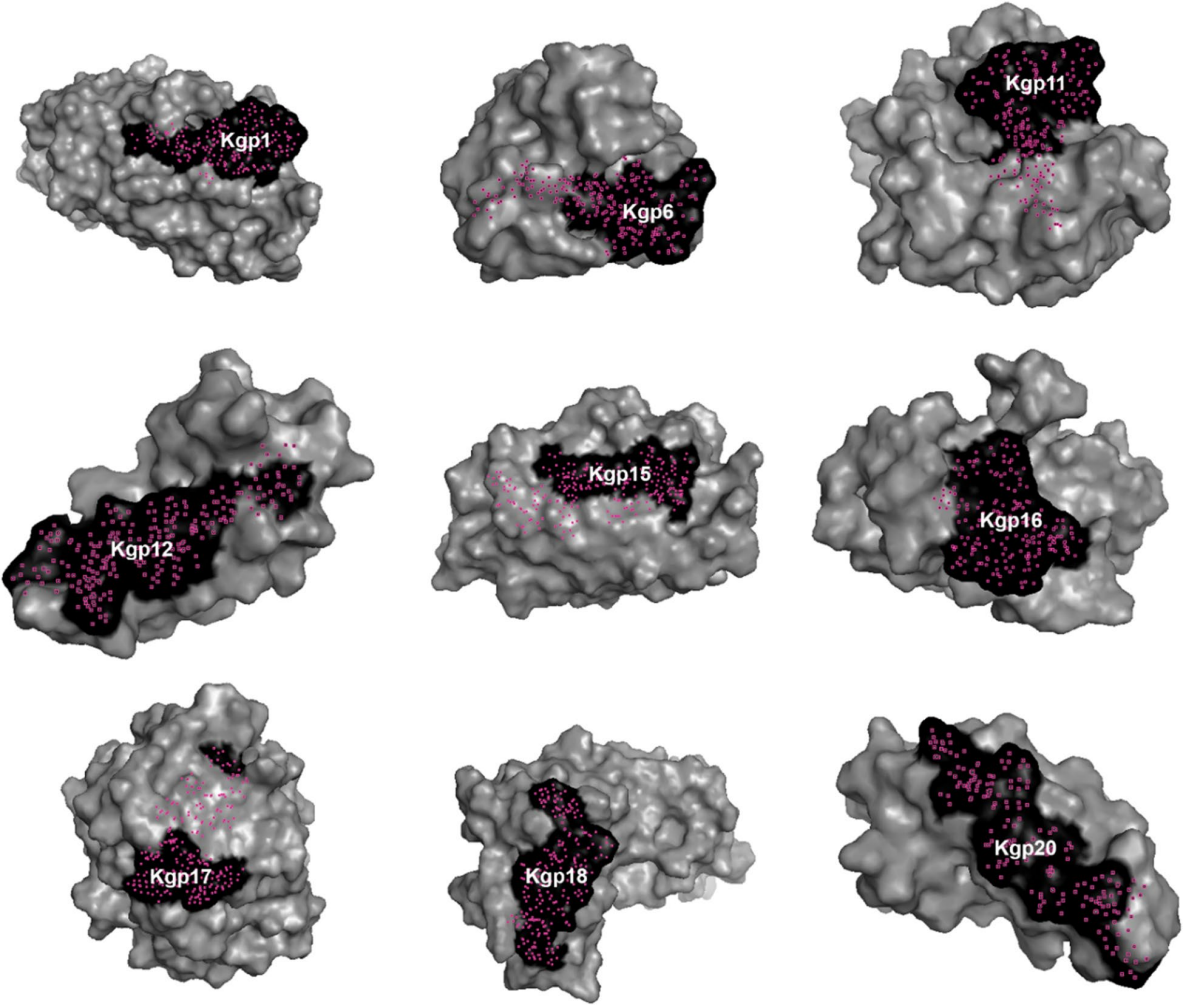
Kgp peptides. Kgp 1, 6, 11, 12, 15, 16, 17, 18 and 20 are schematically presented in black in their protein region after its modeling. Brazil, 2018

**Fig. 3:**
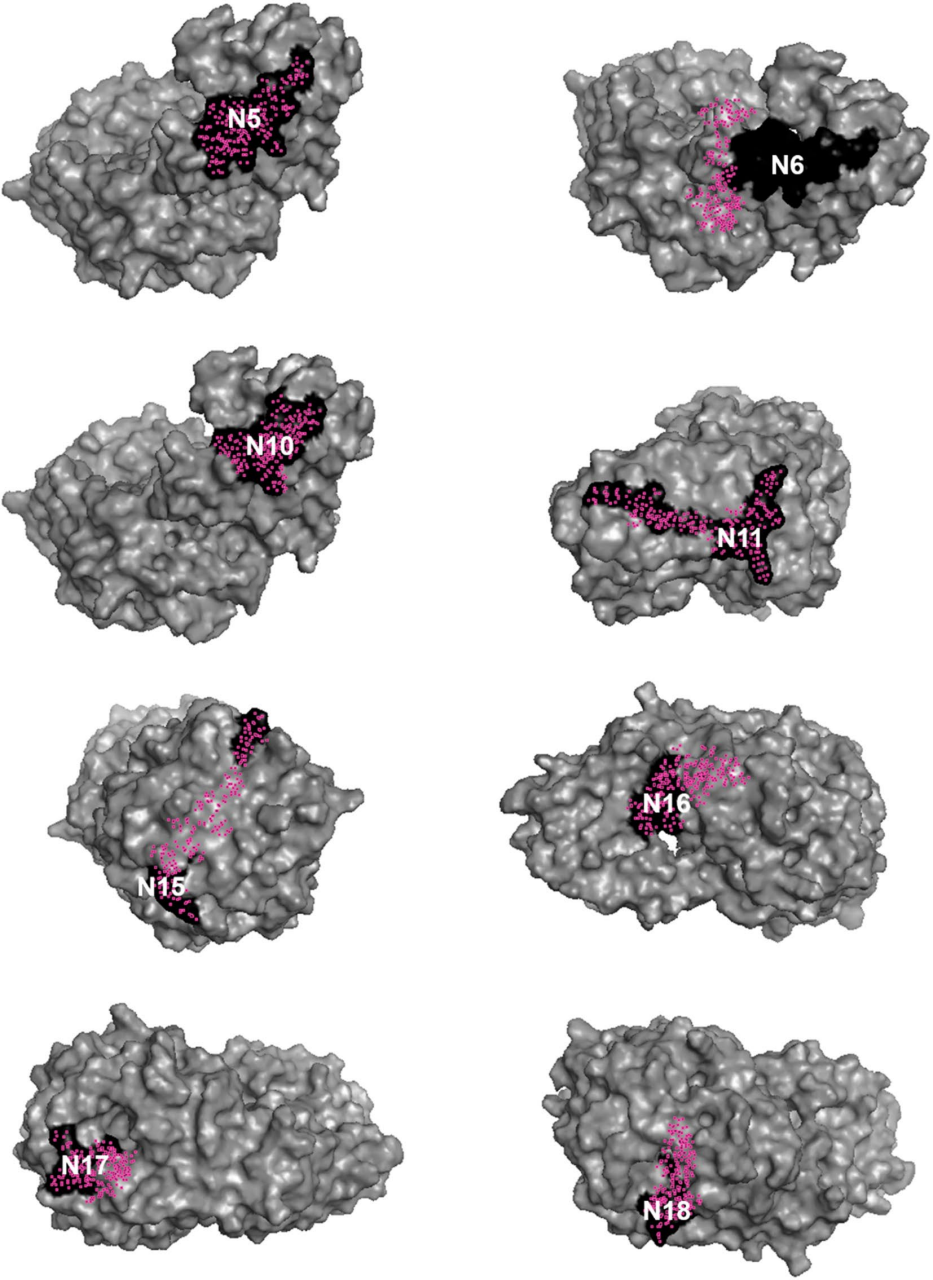
Neuraminidase peptides. N5, N6, N10, N11, N15, N16, N17 and N18 are schematically presented in black in the neuraminidase protein after its modeling. Brazil, 2018

**Fig. 4:**
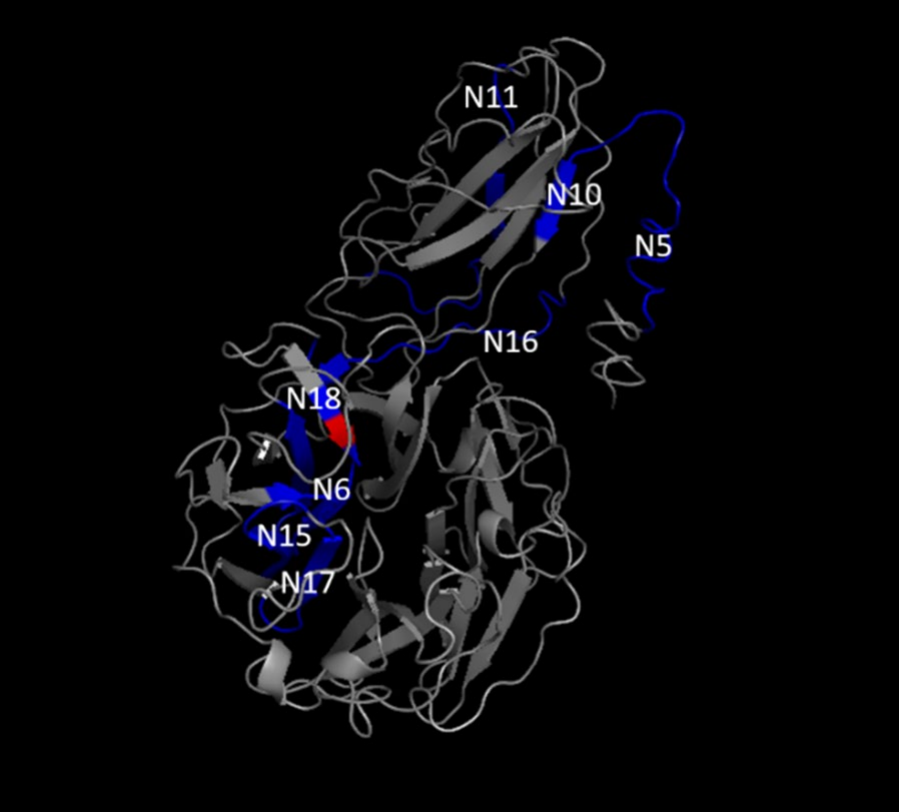
Neuraminidase peptides. N5, N6, N10, N11, N15, N16, N17 and N18 are schematically presented in blue in the protein structure after its modeling. N18 includes the catalytic site presented in red. Brazil, 2018

The published peptides were presented in 3D models shown in surface mode (Figs. 2, 3) and in cartoon mode (Fig. 4). It was not possible to present the entire Kgp protein (1723 aa) in cartoon mode because the tool used for modeling allows one to model up to 1500 amino acid residues.

Peptides from different regions of the proteins were chemically synthesized, tested and published. Figure 4 shows the relationship between neuraminidase peptides. It was not possible to present the entire Kgp protein, but we can provide an overview of Kgp peptides within the Kgp protein in Additional file 1: Table S2.

## Discussion

It is known that in silico models are used for understanding biological systems as well as to select, to complement, and to inspire the required laboratory experiments (Kollmann and Sourjik 2007; Setty 2014; Brodland 2015). In this context, immunoinformatics brings advances in immunology and can contribute to understanding the immune response (Lefranc 2014; Qiu et al. 2018). In the present study, the in silico analysis enabled the prediction and selection of immunoreactive peptides of *P. gingivalis* before being synthesized. Two virulence factors of *P. gingivalis* were analyzed: Kgp, which is widely studied, and neuraminidase, which is still being evaluated in a few studies.

The same HLA alleles were used to predict immunogenic peptides of virulence factors. The HLA alleles tested with neuraminidase peptides were also tested with Kgp peptides and the same pools of sera were used for the immunoreactivity test of the synthetic peptides. However, all tested Kgp peptides presented immunoreactivity to IgG, whereas neuraminidase peptides presented low immunoreactivity to IgG.

Besides that, Kgp12 distinguished between CP and WP pools. The coefficient obtained from the fraction CP pool/WP pool indicated that the absorbance of the CP pool was twice as high as that of the WP pool. Kgp12 was tested in another study to detect specific serum IgG of 71 subjects, and it distinguished the ones with gingivitis from those with chronic periodontitis (Cardoso, unpublished data, 2017).

All tested Kgp peptides presented immunoreactivity to IgG, whereas neuraminidase peptides presented low immunoreactivity to IgG and no neuraminidase peptide presented a coefficient between CP and WP sera that could differentiate subjects with CP; thus the low IgG reactivity of those neuraminidase peptides could be suggested. However, further studies need to be conducted to better define this characteristic of neuraminidase peptides tested herein.

*Porphyromonas gingivalis* extract and gingipains have the capability to induce immunogenicity since they are recognized by IgG from serum of individuals with chronic periodontitis (O’Brien-Simpson et al. 2000; Inagaki et al. 2003; Nguyen et al. 2004; Franca et al. 2007; Trindade et al. 2008, 2012a). The IgG-mediated response in humans to neuraminidase (sialidase) of *P. gingivalis* remains, to our knowledge, unknown. However, in the present study, based on the in silico analysis of tested peptides, under the conditions studied, the IgG reactivity of Kgp could be reaffirmed and the low reactivity of neuraminidase could be speculated.

Besides being asaccharolytic, *P. gingivalis* probably does not use *N*-acetylneuraminic acid (Neu5Ac), the most studied sialic acid, as a nutrient. This deduction was made because in the culture supplemented with Neu5Ac, it did not interfere with *P. gingivalis* planktonic growth, and the inactivation of the neuraminidase gene did not influence its growth (Li et al. 2012). Additionally, not only *P. gingivalis* but also other periodontopathogens, *Tannerella forsythia* and *Treponema denticola*, perform glycosylation of their proteins to evade the immune response, persist in the host and cause periodontal destruction (Stafford et al. 2012; Kurniyati et al. 2013; Settem et al. 2013). This is one reason why the low immunogenicity of its neuraminidase should be a favorable characteristic to *P. gingivalis*, since this virulence factor may be used to evade immune system inflammatory responses.

We also need to consider that, despite being primarily an extracellular pathogen, *P. gingivalis* has the ability to internalize non-phagocytic cells of the host (Nakagawa et al. 2006; Olsen and Progulske-Fox 2015) and to survive in a macrophage (Gmiterek et al. 2016; Yang et al. 2018) in order to evade the immune system. As in the case of other pathogens (Shtyrya et al. 2009; Banerjee et al. 2010; Freire-de-Lima et al. 2015), sialidase may be used by *P. gingivalis* as one of the cellular invasion mechanisms.

The ATCC 33277 strain of *P. gingivalis* (NCBI Taxonomy ID: 431947) is used for pathophysiological characterization of the microorganism, and it is considered a less virulent strain (Naito et al. 2008). Its genome was published by Naito et al. (2008), and the sequences of its proteins and its peptide epitopes have been deposited in online public databases. Epitopes from other antigens of this strain had already been tested and deposited at the IEDB. For example, Kgp peptide DKYFLAIGNCC (Epitope detailed search: Epitope ID 190728) induced IL-17A e IFN-γ production in *Mus musculus* C57BL/6 (Bittner-Eddy et al. 2013). On the other hand, in the IEDB, epitopes related to neuraminidase (sialidase) of *P. gingivalis* were not identified until our deposition. In the present study, eight neuraminidase peptides of *P. gingivalis* were originally deposited in the IEDB, as well as nine novel Kgp peptides, and they are available for public visualization and for use in other assays.

A limitation of the study is that binding to MHC is necessary but not sufficient for epitope recognition by T cells. However, the use of in silico analysis for prediction of immunogenic peptides allowed their selection for chemical synthesis and immunogenicity tests.

*Porphyromonas gingivalis* manipulates the host immune response using the diversity of its virulence factors (Hajishengallis and Lamont 2014; Dashper et al. 2017). The peptides tested herein could be useful for application in immunogenic studies of virulence factors, since it is more economical to use synthetic peptides than to use recombinant proteins. In addition, specific peptides decrease the risk of cross reactivity in the opposite way of the total extract of the microorganism.

The strategy used herein reduced the total analysis cost and the research expended time in the search for promising results. It could be applicable to a global understanding of *P. gingivalis* pathogenicity (as well as other periodontal pathogens) in an efficient and rapid manner using chimeric peptides as well. Thus, in silico tools provide comprehensive facilities for designing in vitro and in vivo immunology experiments. This can be a useful strategy in the study of the etiology and pathogenesis of human diseases, especially periodontitis.

In conclusion, here we report the identification of *P. gingivalis* epitopes. In silico analysis represented a viable strategy to obtain candidate epitopes with human IgG reactivity to study *P. gingivalis* virulence factors, because the IgG reactivity of Kgp could be reaffirmed and the low reactivity of neuraminidase could be suggested. Kgp12, Kgp17 and Kgp18 peptides were selected for subsequent assays. Besides that, the present study has provided immunogenic peptide sequences from *P. gingivalis* virulence factors, which may be tested by several different assays in order to contribute to the understanding of the host response to infection and to differentiate subjects with chronic periodontitis.

## Additional file

**Additional file 1.** Additional Figure and Tables.
