# Supplementary material for: In silico analysis as a strategy to identify candidate epitopes with human IgG reactivity to study Porphyromonas gingivalis virulence factors
Source: AMB Express. 2019 Mar 11;9:35. doi: 10.1186/s13568-019-0757-x (PMC6411804; doi:10.1186/s13568-019-0757-x)
Supplement: Supplementary file 1 — Additional file 1. Additional Figure and Tables. [file 13568_2019_757_MOESM1_ESM.pdf]

AMB Express

In silico analysis as a strategy to identify candidate epitopes with human IgG reactivity to study *Porphyromonas gingivalis* virulence factors

Ellen Karla Nobre dos Santos-Lima[1], Kizzes Araújo Paiva Andrade Cardoso[2], Patrícia Mares de Miranda[1], Ana Carla Montino Pimentel[1], Paulo Cirino de Carvalho-Filho[3], Yuri Andrade de Oliveira[4], Lília Ferreira de Moura-Costa[5], Teresa Olczak[6], Isaac Suzart Gomes-Filho[7], Roberto José Meyer[5], Márcia Tosta Xavier[3], Soraya Castro Trindade[7].

[1]Postgraduate Program in Immunology, Federal University of Bahia, Salvador, Bahia, Brazil; [2]Biotechnology Postgraduate Program, Federal University of Bahia, Salvador, Bahia, Brazil; [3]Bahian School of Medicine and Public Health, Salvador, Bahia, Brazil; [4]Dentistry Course, Feira de Santana State University, Feira de Santana, Bahia, Brazil; [5]Department of Biointeraction, Federal University of Bahia, Salvador, Bahia, Brazil; [6]Faculty of Biotechnology, University of Wrocław, Wrocław, Poland; [7]Department of Health, Feira de Santana State University, Feira de Santana, Bahia, Brazil.

[1,2,5]Avenida Reitor Miguel Calmon s/n, Vale do Canela, Salvador, Bahia, CEP 40110-100, Brasil.

[3]Av. Silveira Martins, 3386, Cabula, Salvador, Bahia, CEP 41150-100, Brasil.

[4,7]Avenida Transnordestina s/n, Novo Horizonte, Feira de Santana, Bahia, CEP 44036-900, Brasil.

[6]F. Joliot-Curie 14A St., 50-383, Wrocław, Poland.

Correspondence

Soraya Castro Trindade

Email: soraya@uefs.br

Table S1: HLA alleles used for prediction of peptidic epitopes at MHC-II Binding Predictions tool from the Immune Epitope Database (IEDB). Brazil, 2018

HLA allele*	HLA allele / haplotype available in the prediction tool	HLA allele / haplotype evaluated in the prediction tool (selected by a consensus approach**)	HLA allele / haplotype used for prediction (consensus method**)
DQB1*03:01	DQA1*05:01/DQB1*03:01	DQA1*05:01/DQB1*03:01	DQA1*05:01/DQB1*03:01
DQB1*02	DQA1*05:01/DQB1*02:01	DQA1*05:01/DQB1*02:01	DQA1*05:01/DQB1*02:01
DQB1*06	DQA1*01:02/DQB1*06:02	DQA1*01:02/DQB1*06:02	DQA1*01:02/DQB1*06:02
DRB3*	DRB3* 01 (01:01, 01:04, 01:05, 01:08, 01:09, 01:11, 01:12, 01:13, 01:14)<br>DRB3* 02 (02:01 - 02:25)<br>DRB3* 03 (03:01, 03:03)	DRB3*01:01<br>DRB3* 02:01<br>DRB3*03:01<br>DRB3*03:03	DRB3*01:01
DRB1*13	DRB1*13 (13:01-13:101)	DRB1*13:01<br>DRB1*13:02	DRB1*13:02
DRB1*15	DRB1*15 (15:01-15:49)	DRB1*15:01 - 15:05	DRB1*15:01
DRB1*07	DRB1* 7 (07:01-07:19)	DRB1*07:01	DRB1*07:01
DRB5*	DRB5* (01:01-01:14)<br>DRB5* (02:02-02:05)	DRB5*01:01<br>DRB5*02:02	DRB5*01:01
DRB4*	DRB4* (01:01-01:08)	DRB4*01:01	DRB4*01:01

*Monteiro (2017). **Predictions are limited to alleles that are currently covered by specific prediction methods.

Table S2: Kgp peptides annotation: putative immunogenic Kgp peptides and their situation within the protein. Brazil, 2018

Protein	Peptide	Start	End	Protein Region - NCBI Protein Database (YP_001929844.1 / WP_012458488.1)						
				**Propeptide C25 (20-226)**	**Peptidase C25 family N-terminal domain (235-595)***	**Peptidase C25 family C-terminal domain (600-679)****	**Domain of unknown function (716-877)**	**Cleaved Adhesin (984-1149)**	**Cleaved Adhesin (1158-1329)**	**Cleaved Adhesin (1424-1589)**
	kgp1	1028	1042					X		
	kgp2	1467	1481							X
	kgp3	1137	1151					X		
	kgp4	1533	1547							X
	kgp5	160	174	X						
	kgp6	1474	1488							X
	kgp9	1317	1331						X	
Kgp (1723 aa)	kgp10	1579	1593							X
	kgp11	733	747				X			
	kgp12	661	675			X				
	kgp14	1	15							
	kgp15	1128	1142					X		
	kgp16	773	787				X			
	kgp17	243	257		X					
	kgp18	136	150	X						
	kgp20	670	684			X				

*Gingipain subgroup of the Peptidase C25 family N-terminal domain. **Peptidase family C25, C terminal ig-like domain. Catalytic sites residues: 442-445,475,477.

Table S3: Neuraminidase peptides annotation: putative immunogenic neuraminidase peptides and their situation within the protein. Brazil, 2018

Protein	Peptide	Start	End	Protein Region - NCBI Protein Database (BAG34127.1)		
				Region name "Sialidase" (182-517)	BNR repeat-like domain. BNR 2* (204-500)	catalytic site (active)**
Neuraminidase (526 aa)	N1	268	282	X	X	
	N2	116	130			
	N3	496	510	X	X	504
	N4	510	524	X		
	N5	17	31			
	N6	199	213	X	X	
	N7	488	502	X	X	488
	N8	98	112			
	N9	83	97			
	N10	22	36			
	N11	65	79			
	N12	200	214	X	X	
	N13	195	209	X	X	
	N14	257	271	X	X	
	N15	263	277	X	X	
	N16	170	184	X		
	N17	254	268	X	X	
	N18	193	207	X	X	194

*BNR: Bacterial Neuraminidase Repeat. This family of proteins contains BNR-like repeats suggesting these proteins may act as sialidases. **Catalytic sites residues: 194, 219, 382, 398, 460, 488 and 504. Among the putative neuraminidase immunogenic peptides,10 were related with the BNR repeat-like domain and 03 of these included a catalytic site.

Table S4. HLA alleles tested with Kgp and neuraminidase peptides in the immunoreactivity test. Brazil, 2018

HLA alleles / haplotype used for prediction	HLA alleles tested with Kgp peptides	HLA alleles tested with neuraminidase peptides
DQA1*05:01/DQB1*03:01	X	
DQA1*05:01/DQB1*02:01		
DQA1*01:02/DQB1*06:02	X	X
DRB3*01:01		
DRB1*13:02	X	
DRB1*15:01	X	X
DRB1*07:01	X	X
DRB5*01:01	X	X
DRB4*01:01	X	X

Table S5. The population coverage of Kgp peptides through the population coverage tool from Immune Epitope Database (IEDB). Brazil, 2018

Peptide	HLA allele	HLA allele genotypic frequency (%)	Population Coverage MHCII (%)
Kgp1	DQB1*03:01	Brazil 33.94<br>South America 27.96<br>North America 25.02<br>World 20.20	Brazil 51.71<br>South America 41.70<br>North America 39.67<br>World 33.44
Kgp6	DQB1*06:02	Brazil 2.75<br>South America 5.00<br>North America 9.72<br>World 7.78	Brazil 4.88<br>South America 8.28<br>North America 16.59<br>World 13.68
Kgp11	DRB1*13:02	Brazil 1.96<br>South America 2.44<br>North America 4.24<br>World 3.81	Brazil 3.54<br>South America 3.78<br>North America 8.30<br>World 6.69
Kgp12	DRB1*13:02	Brazil 1.96<br>South America 2.44<br>North America 4.24<br>World 3.81	Brazil 3.54<br>South America 3.78<br>North America 8.30<br>World 6.69
	DRB1*15:01	Brazil 1.91<br>South America 3.37<br>North America 12.13<br>World 10.82	Brazil 3.46<br>South America 5.20<br>North America 22.79<br>World 18.41
Kgp12	DRB1*13:02<br>DRB1*15:01	-	Brazil 6.94<br>South America 8.88<br>North America 30.06<br>World 24.44
Kgp15	DRB1*07:01	Brazil 6.01<br>South America 6.10<br>North America 10.21<br>World 10.71	Brazil 10.67<br>South America 9.31<br>North America 19.38<br>World 18.23
Kgp16	DRB1*07:01	Brazil 6.01<br>South America 6.10<br>North America 10.21<br>World 10.71	Brazil 10.67<br>South America 9.31<br>North America 19.38<br>World 18.23
Kgp17#	DRB5*01:01	Allele was not available	-
Kgp18#	DRB5*01:01	Allele was not available	-
Kgp20#	DRB4*01:01	Allele was not available	-
**Epitope set**	All alleles tested#	-	Brazil 62.82<br>South America 57.22<br>North America 74.33<br>World 66.80

DQ and DR available alleles: DQA1, DQB1 and DRB1. # It was not included in the epitope set. South America: Brazil, Chile, Peru, Colombia, Paraguay, Argentina, Bolivia, Venezuela, Ecuador. North America: Canada, United States, Mexico. Population datasets provided by The Allele Frequency Net Database. Accessed May 21, 2018.

Table S6. The population coverage of neuraminidase peptides through the population coverage tool from Immune Epitope Database (IEDB). Brazil, 2018

Peptide	HLA allele	HLA allele genotypic frequency (%)	Population Coverage MHC II (%)
N5	DQB1*06:02	Brazil 2.75<br>South America 5.00<br>North America 9.72<br>World 7.78	Brazil 4.88<br>South America 8.28<br>North America 16.59<br>World 13.68
N6	DQB1*06:02	Brazil 2.75<br>South America 5.00<br>North America 9.72<br>World 7.78	Brazil 4.88<br>South America 8.28<br>North America 16.59<br>World 13.68
N10[#]	DRB4*01:01	Allele was not available	-
N11[#]	DRB5*01:01	Allele was not available	-
N15	DRB1*15:01	Brazil 1.91<br>South America 3.37<br>North America12.13<br>World 10.82	Brazil 3.46<br>South America 5.20<br>North America 22.79<br>World 18.41
N16	DRB1*15:01	Brazil 1.91<br>South America 3.37<br>North America 12.13<br>World 10.82	Brazil 3.46<br>South America 5.20<br>North America 22.79<br>World 18.41
N17	DRB1*07:01	Brazil 6.01<br>South America 6.10<br>North America 10.21<br>World 10.71	Brazil 10.67<br>South America 9.31<br>North America 19.38<br>World 18.23
N18	DRB1*07:01	Brazil 6.01<br>South America 6.10<br>North America 10.21<br>World 10.71	Brazil 10.67<br>South America 9.31<br>North America 19.38<br>World 18.23
**Epitope set**	All alleles tested[#]	-	Brazil 18.13<br>South America 21.36<br>North America 49.70<br>World 43.71

DQ and DR available alleles: DQA1, DQB1 and DRB1. [#] It was not included in the epitope set. South America: Brazil, Chile, Peru, Colombia, Paraguay, Argentina, Bolivia, Venezuela, Ecuador. North America: Canada, United States, Mexico. Population datasets provided by The Allele Frequency Net Database. Accessed May 29, 2018.

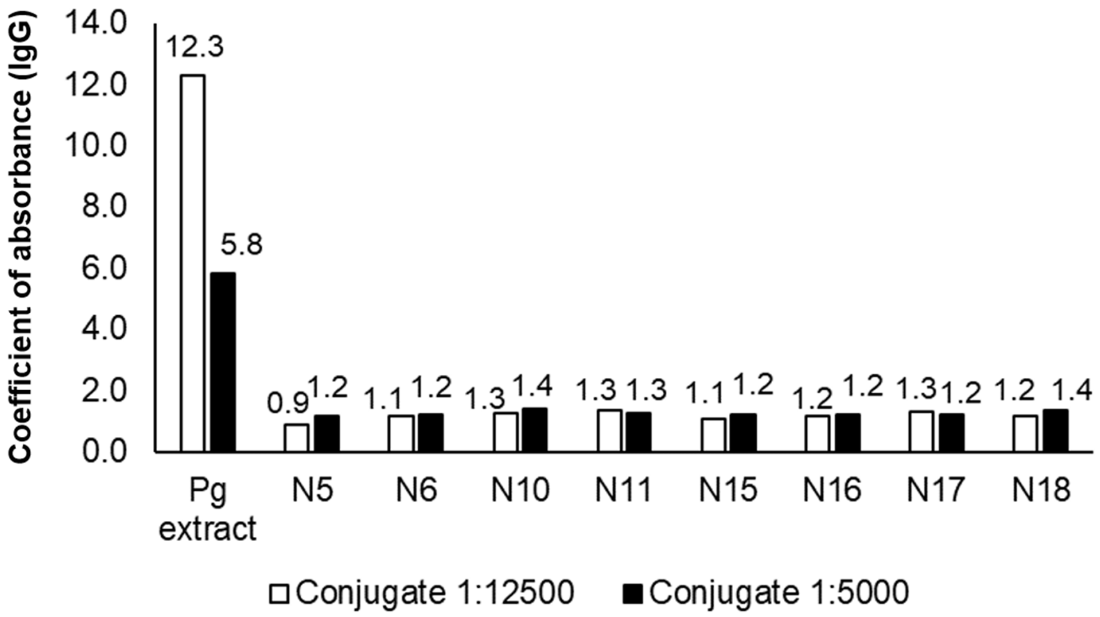

Figure S1. Coefficients of absorbance between CP sera pool and WP sera pool after checkerboard ELISA analysis of neuraminidase synthetic peptides. Brazil, 2018

CP: Chronic Periodontitis, WP: Without Periodontitis. The coefficient is the difference of the O.D. value between the sera pools and it expresses the difference of the mean IgG levels between the CP and the WP sera pools. *P. gingivalis* (Pg) extract and peptides were used as antigen in indirect ELISA test. Anti-human IgG peroxidase conjugate dilution 1:12500 and 1:5000 are represented. None peptide presented a satisfactory coefficient between CP and WP sera pools. Checkerboard ELISA condition represented: 5µg/mL Pg extract concentration, 10 µg/mL peptide concentration, 1:100 sera pool concentration and 1:12500 / 1:5000 anti-human IgG peroxidase conjugate concentration. The nature of data does not allow statistical analysis between the coefficients.
